# Demonstration of a Home Laundering Method for Cloth Facepieces to Achieve Hygienic and Sustainable Reuse

**DOI:** 10.1177/10482911251334843

**Published:** 2025-05-08

**Authors:** William Mackay, Chris Baglin, Paul Baglin, Claire Chalmers, Fiona Henriquez, Ngozi Amaeze

**Affiliations:** 1 School of Health and Life Sciences, 6413 University of the West of Scotland, Glasgow, UK; 2 tensARC, Stirling, UK; 3 Department of Civil & Environmental Engineering, University of Strathclyde, Glasgow, Scotland, UK

**Keywords:** laundering, reprocessing, reusable, facepiece, face mask

## Abstract

Mask shortages during COVID-19 led to the adoption of reusable textile masks; research into their performance and optimal washing conditions can guide domestic laundering to encourage their use, decreasing plastic pollution. The study tested four washing methods for cleaning artificially contaminated facepieces. These conditions included nonbiological detergent at 30°C, Reference Detergent 3 (RD3) at 40°C and 60°C, and fortified RD3 (sodium perborate + tetraacetylethylenediamine) at 40°C. After washing, the facepieces were tumble- or air-dried. The effectiveness was determined by measuring bacteria reduction by standard plate count, achieving a target reduction of ≥99.99% and a benchmark cleanliness requirement (for surgical masks) of ≤ 30 CFU/g (EN 14683: 2019). All met the benchmark except 30°C nonbiological detergent washes with air drying. Oxidative bleach reduced RD3 performance. This research demonstrates that heavily contaminated reusable masks can be effectively decontaminated using domestic machines on a normal wash cycle (40 degrees).

## Introduction

Hazards presenting potential risks to the human respiratory system arise in many settings and circumstances. Manufacturers design facepieces to protect the wearer from hazards, to protect others from hazards expelled by the wearer, or both. For example, some facepieces are designed for the purpose of limiting exposure to dust particles and others for the purpose of limiting the risk of respiratory disease transmission by serving as a barrier to the wearer's respiratory particles being exhaled or inhaled by others. Facepieces can function as a risk mitigation tool, when designed, worn, and cared for properly. Given the variety of hazards, individuals may wear facepieces for many different reasons, such as to control infection in medical settings,^1^ to stop the spread of transmissible respiratory diseases in the community,^2^ to reduce the impacts of cold temperatures,^3^ to minimize allergies,^4^ and to mediate particulates in the environment stemming from pollution,^5^ wildland fires,^6^ volcanoes,^7^ flood damage,^8^ dried-up water bodies,^9^ nuisance dust,^10^ and other hazards. Using a cloth facepiece in any of these situations raises questions about its reuse over time.

When a worn facepiece is no longer effective, it needs replacement or reprocessing to a usable condition. Disposable facepieces are designed to be discarded after a single or limited use,^11^ although studies suggest some types, including medical or surgical masks^12^ and filtering facepiece respirators,^13^ can be reprocessed and used again on a limited basis. Other facepieces are designed and intended for reuse; and, accordingly, they will be manufactured to relevant standards, if available, such as those standards applicable to reusable elastomeric respirators and barrier face coverings.^14^

In times of necessity or shortage, improvised facepieces are an alternative option for the community.^15^ Manufacturers have relied on the physical and behavioral sciences, including aerosol science, textile science, microbiology, and ergonomics, to design, develop, and manufacture at-scale, reusable facepieces to meet community needs. For example, a consensus developed during the COVID-19 pandemic that multiple layers of fabric are a useful cloth facepiece design, and this configuration is supported by various authorities and experts.^16^ Guidance from the American Association of Textile Chemists and Colorists (AATCC) suggests specific fabrics for various mask layers, as seen in Table S1, AATCC M14-2020, Guidance and Considerations for General Purpose Textile Face Coverings: Adult. Further, studies confirm certain fabrics, such as polyester, are effective for cloth facepieces^17^; however, bioaerosols bind more easily to some fabrics, including polyester^18,19^ making it important for manufacturers to validate the effectiveness of care and washing instructions.

Government or organizational policies may call for the wearing of facepieces, and employers are to assist affected employees in their use in the workplace consistent with relevant rules and expert guidance. Employers can direct the type and model(s) of disposable or reusable facepieces to wear, for example. In contrast, outside of occupational settings there may be no requirement for (or availability of) expert assistance when donning, doffing, or cleaning facepieces; their use—and reuse—in the community is likely to be unassisted. Therefore, a facepiece designed for reuse, as well as its technical information, not only should help remove the target hazard (e.g., pathogen, nuisance dust or other hazard in the environment) but also provide simple and effective support for the wearer's unassisted use and reprocessing of the product as well as mitigate the risk of degradation from repeated wear and reprocessing. When designing reusable facepieces, manufacturers have turned to launderable textiles and fabrics, and yet, while manufacturer instructions or public health guidance may be available, there are few details on the use and care of reusable cloth facepieces in the community. Domestic laundering in particular lacks targeted guidance or standards.

Environmental health hazards are a persistent risk in the community, and they also can be present in occupational settings when controls are reduced. Conditions can lead to improvised applications of facepieces^7,20^ and accessories^21,22^ for protection from respiratory hazards. During the COVID-19 pandemic, global use of facepieces drew attention to their limitations, as well as the limitations in extending the traditional varieties of facepieces to uses that are new or not fully validated. The Committee on Respiratory Protection for the Public and Workers Without Respiratory Protection Programs at Their Workplaces at the National Academic of Sciences concluded that communities need new options and guidance for their unassisted use of facepieces.^6^ To advance beyond initial questions about the immediate effectiveness of current facepiece solutions, it is helpful to consider the concept of sustainability, a principle which places economic, environmental, and social concerns on equal footing.^23^

With respect to economics, the use of disposable products creates supply chain issues when demand increases rapidly in an emergency. Infectious disease epidemics and pandemics lead to increased use of facepieces,^24,25^ and wide-spread adoption of disposable face masks generally over the past century^26^ introduced a risk of potential supply shortages during high-demand periods.^27,28^ Use of respirators as an emergency response, for example, will cause supply chain issues due to the sheer volume required (a minimum of one per person per day). Spikes in demand produce disruptions in logistics, manufacturing, and parts of the economy. On the other hand, society's long-term reliance on disposable facepieces has resulted in a set of standards for assessing their performance with respect to health and safety. Among these standards, for example, is *BS EN 14683:2019, Medical face masks. Requirements and test methods*, which states the bioburden permissible for a new, disposable medical face mask, and offers a benchmark helpful to manufacturers’ economic objectives.

With respect to environmental concerns, recent experience exemplifies a core problem. Litter from disposable facemasks increased with the increase in facemask usage during the COVID-19 pandemic, with one analysis of 11 countries finding a more than 80-fold increase in such litter.^29^ In addition to solid waste concerns, disposable masks present pollutant issues. Plastic waste, undergoing physical, chemical, and biological degradation processes, has infiltrated both terrestrial and aquatic ecosystems, releasing heavy metals, volatile organic compounds, and microfibers.^30^ Due to their composition, plastics persist in the environment,^31^ creating a potential threat to ecosystem and human health.^32,33^ Despite these issues, disposable face masks made of polymeric materials remain popular in healthcare and in the community.^34,35^

With respect to social concerns, disposable facepieces may give rise to social inequities when a community at risk of exposure to an environmental health hazard must “make do” with choices among products which are in short supply, do not fit, or are otherwise unsuitable despite the convenience of being disposable.

Reusable facepieces provide a viable solution from an economic perspective. Reusable cloth face masks have been shown to be effective compared to no face masks in reducing exposure to influenza virus^36^ and are capable of filtering droplets^37^ and aerosols.^38^ A cloth face mask may reduce exposure to airborne respiratory hazards.^39,40^ A reusable cloth facepiece also can be useful in rugged environments.^3^ Cloth facepieces can be washed and air dried without reducing performance, although the impact of washing will depend on the yarn type and construction.^12^ Reusability adds to the economic benefit of cloth masks.^41^ Reusable face coverings can shield the wearer from supply issues, because they can be worn multiple times with proper care, including cleaning and maintenance.

The economic sustainability of reusable facepieces is dependent in part on the presence of industry standards, and the textile and apparel industries have multiple points of reference for manufacturers and consumers of reusable facepieces (see Supplemental file for details).

As with AATCC, the COVID-19 pandemic spurred a variety of international authorities to develop guidance on the cleaning of used masks, including details useful to manufacturers and to the user in a community setting. Supplemental Table S1 provides a summary of such guidance, whose many examples address cleaning by hand or by machine at various temperature levels and provide guidance on drying with and without a machine dryer. These forms of guidance to industry and consumers have limitations. Typically, the guidance is brief and at a very high level, and the presence or absence of some interventions, for example, machine drying, vary.

A minimum washing temperature is a common recommendation. Supplemental Table S1 illustrates that washing temperature has been a key variable in guidance stemming from the COVID-19 pandemic, with temperatures ranging from 40°C to 60°C to 70°C depending on other conditions The examples of guidance in Supplemental Table S1 sometimes do not capture the variability in laundering conditions or contexts reported in studies (see Supplemental file for further details on specific temperature guidance). Recent analysis of various facepiece studies concludes that 60°C is a preferred washing temperature under lab conditions.^28^ As noted, studies suggest higher temperatures, but given human factors, including uncertain personal resources and behavior, these high-temperature conditions may not be practical for domestic settings. Research on home laundering conditions and the reprocessing of textile-based PPE suggests laundering at home may not be sufficient for mitigating pathogen risks.^42^ One set of investigators observed that nurses preferred laundering uniforms at temperatures at 40°C which was viewed as potentially not sufficient to achieve decontamination and cleanliness.^43^ A recent study found that 17% of healthcare worker respondents did not use the recommended temperature for home laundering of their worn uniforms.^44^

Prior studies have tested textiles generally and, more specifically, cloth facepieces designed for reuse. In both cases, their methods and results may be challenging for a facepiece designer and manufacturer to translate into technical guidance supporting replicable manufacturing and testing processes. Studies may assess the relative reduction in pathogen levels by various methods^45^ but there is no widely accepted performance level or other benchmark level of cleanliness, nor a decontamination standard, to which manufacturers can refer in developing reusable face pieces and testing them.

With respect to the environmental prong of sustainability, reusable facepieces can help reduce litter and mitigate other issues, such as releases of chemicals and microplastics, that are associated with single use facepieces. Impacts, small or large, from laundering reusable facepieces also deserve consideration. Due to environmental concerns and market demands, detergent manufacturers have increasingly developed products that clean at reduced temperatures; to achieve effectiveness at lower temperatures, contemporary detergents typically incorporate a bleach activator.^46^ In addition to enhancing washes at colder temperatures, additives such as oxidative bleach in laundry detergents offer benefits over standard soap such as improved cleaning and biodegradable formulations. Sodium perborate, an economical and stable oxidizing agent, undergoes hydrolysis during use, producing hydrogen peroxide and borate. Hydrogen peroxide is fully decomposed through reactions with reducing substances in the washing liquid; borate, on the other hand, remains in the wastewater where it exhibits low toxicity to plants and fishes.^47^ Other chemical compositions such as tetraacetylethylenediamine (TAED) exhibit excellent biodegradability and are largely removed during sewage treatment; as a result, TAED presence in the aquatic environment remains minimal.^48^

With respect to social sustainability, reusable cloth facepieces implicate certain social issues, including health. Used cloth products, including facepieces, may collect and hold a bioburden of potentially harmful levels of pathogens.^49–53^ An institutional setting may use controls to address the risk from contaminated cloth materials, such as their isolation in a designated laundry bin or wash bag. It must be asked if society is sufficiently informed to handle and reprocess reusable cloth facepieces (containing a bioburden of pathogens from the wearer and the environment) within the community and whether current guidance helps to maintain their safe and effective use. Supplemental Table S1's guidance documents were largely published under the emergency conditions of the COVID-19 pandemic and as such have limitations. Limitations include little guidance on when health and safety require retirement of a given facepiece or the removal of dirt, sweat, pathogens, toxic chemicals, or other contaminants prior to reuse. Further, inadequate reprocessing of reusable face masks is not only a concern when wearing the masks themselves but also poses a risk of cross-contamination within the entire wash load. This cross-contamination can potentially spread pathogens and contaminants to previously clean materials and risk compromising the hygiene of the entire laundry batch. Some of the guidance collected and presented in Supplemental Table S1 is explicit about using detergent, potentially because the guidance emerged during the COVID-19 public health emergency to address SARS-CoV-2 specifically, which was found to have low survival when a detergent is used.^54,55^

Social considerations can help place more emphasis on subjective comfort, in addition to facepiece functionality such as filtration level and other performance metrics. In Krishnan et al.,^56^ investigators considered comfort to the wearer to be a key variable, encouraging user tests on harness strength, seams, stitching, breathing comfort, and moisture issues, to assess the best fit over time.

Managing the inspection of reprocessed facepieces can differ by setting. European standards, also noted in Krishnan, call for visual inspection after each wash for no defects, such as tears, detachments, deformation, or wear of materials, in addition to inspecting for a looser fit.^57^ Such visual inspection can be subjective and may be conducted in an unassisted setting, creating uncertainty regarding its reliability. This “human factor” may raise potential social equity concerns when proper instruction is not provided and may distinguish the use of facepieces in the community from use in regulated occupational settings. For example, the outward appearance of a textile can influence how an individual perceives its effectiveness and therefore influence whether it is worn. Esthetic considerations include wrinkle resistance, pilling, and colorfastness. Colorfastness in particular is a feature whose degradation may lead the unassisted user to believe the item is no longer effective even when performance testing would indicate full functionality remains. Social considerations also include ensuring diversity in the populations used by manufacturers to gauge fit performance after reprocessing. Krishnan et al.^56^ states: “to ensure an optimal fit, visual fit tests should be conducted on a range of users (head size or age).”

Systematic reviews vary in their conclusions but point to an ongoing concern over the risks of washing, in a domestic setting, those textiles potentially contaminated with microbes prevalent in hospitals.^58^ Given evidence that laundering uniforms at home may not be as reliable as that conducted institutionally, it is important to determine whether variances from 60°C during home laundering of reusable, cloth face pieces create an unacceptable risk.

The preceding list of economic, environmental, and social considerations is long and most likely incomplete, but it suggests that an evidence base and validated practices are needed to advance sustainable reuse of facepieces. Use of reusable facepieces calls for a demonstration that their reprocessing is readily achievable by the wearer, results in a safe product, preserves the performance of the product and is repeatable.

The study discussed in this article is one of several conducted by a team of investigators focused on these goals. The team reviewed the functional requirements and suitable performance levels for reusable cloth facepieces, including those addressed in available standards or emerging standards, such as breathing resistance, filtration, and fit. Filtration, breathing resistance, and fit research results (as well as outcomes from multiple comfort testing studies and the status of data analysis) are reproduced in Supplemental 1 (see Supplemental file), including test results from independent labs confirming these properties and performance levels were maintained after reprocessing.

The data and analysis herein are from a complementary study: an evaluation of the laundering of cloth facepieces intended for reuse. Distinct study objectives included identification of a reprocessing method that can be achieved and repeated as a part of routine laundering at home and can inactivate potential pathogens to reduce bioburdens to generally accepted levels. Another objective was to identify an acceptable absolute cleanliness level based on the values directed for new disposable medical masks. Attaining an absolute level is different from simply proving a relative reduction in bioburden. Meeting this objective would indicate a simpler approach for those manufacturers desiring to test the reprocessing of their products, collect relevant technical information, and thereby inform the care and other instructions that they provide to end users in the community.

## Methods

The study assessed reprocessing outcomes by reference to an existing cleanliness performance standard for new disposable medical masks. The investigators sought to ensure the reprocessing method did not degrade the facepiece. Therefore, the present study used materials and samples from a single facepiece model, detailed in supplementary file, composed of polyester, a robust material recommended for cloth face pieces but to which pathogens bind easily, as noted in the Introduction. The cloth facepiece also proved to have the following attributes: (1) high performance in filtration and breathing resistance as determined through testing by an independent entity before and after extensive reprocessing under similar conditions; (2) high performance in a leakage assessment conducted in an academic setting and whose data has been reviewed and accepted for summary presentation on a government website; and (3) acceptable results from investigations using both objective measures of comfort, noted in supplementary file, in previously reported results and subjective assessments of comfort, to be reported in a forthcoming paper.

The reprocessing method in the present study addressed the potential microbial hazards which may bind to a cloth mask by selecting suitable proxies for use in testing. The swatches used were from a commercially available, reusable cloth facemask (one type of mask and one filter construction) whose sustained performance after reprocessing has been confirmed for filtration, breathing resistance and fit. It was composed of synthetic materials, including a warp knit polyester outer layer, a nylon liner, a polyester fleece filter, and a polyester/elastane harness, as described in Baglin et al.^40^ Test conditions, for example, water temperature, were derived from existing standards for common textiles because they were likely to preserve mask functionality and suit home laundering conditions. To determine the effectiveness of different detergents and temperatures in the decontamination of reusable cloth face masks, a performance benchmark for accepting a wash cycle as adequate was selected. This benchmark followed the bioburden permissible under BS EN 14683:2019 for a disposable medical face mask; as such, the study benchmark for cleanliness was 30CFU (colony forming units)/g.

### Selection and Preparation of Test Organisms

Two bacterial species were used in the study, *Pseudomonas aeruginosa* NCTC 10332 and *Streptococcus mutans* (verified through 16 s sequencing). *P. aeruginosa* NCTC 10332 was purchased from Public Health England culture collections and *S. mutans* was provided by School of Health and Life Sciences, UWS, on tryptone salt agar (TSA) (Oxoid, Basingstoke, UK) plates and verified through 16 s sequencing. Both bacteria were maintained on TSA.

Inoculum preparation and size were prepared following a method adapted from Bockmühl.^59^ A typical colony of each test organism was taken into 10 mL of Tryptone Salt broth, TSB (Oxoid, Basingstoke, UK) and incubated at 37°C in air overnight at rotation speed of 130 rpm in an orbital shaker incubator ES-80 (Cambridgeshire, UK). On the day of the test, 1 mL of each bacterial suspension was taken from the overnight culture into a sterile 1.5 mL Eppendorf tube and centrifuged in a microcentrifuge (Micro Centaur, Scotlab, London, UK) at 5,590xg for 5 min. The bacterial pellet was washed twice with phosphate buffer saline (PBS) (Fisher Scientific, Leicestershire, UK) and the bacterial suspension adjusted to an OD of 0.2 ± 0.02 at 570 nm which was approximately 1 × 10^9^ CFU/mL.

### Preparation of the Face Mask Material Swatches

All face mask material swatches were held in autoclave bags to sterilize by autoclaving and then dried at 40°C to 50°C in a drying cabinet (LEEC FCX1, Nottingham, UK) prior to experimentation and used within a month. The swatches were held in small batches in sterile autoclave bags which were kept open in the drying cabinet to allow free circulation of air and quick evaporation of moisture. The swatches were dried to a constant weight with the holding bags secured to prevent contamination of the swatches after drying.

### The Quantitative Survival Test

The hygienic efficacy of the laundering procedure was assessed by comparing the microbiological counts of the test swatches before and after laundering. Sterile swatches (transfer controls) were washed alongside the bacteria seeded swatches to look for cross-contamination. Dry sterile swatches were removed one at a time using flame-sterilized forceps and placed in the center of a sterile 90 mm petri dish. Contamination of test and control swatches was undertaken with 100 mL of each test organism on each test and control swatch. For uniformity, contamination was maintained on the colored side of each swatch after which they were allowed to dry for 30 min under a class 2 safety cabinet. The contaminated swatches were used within 1 h of drying. Tests were done using three biological replicates and three technical replicates. The controls consisted of swatches contaminated with bacteria and not washed (positive control), swatches not contaminated with bacteria and not washed (negative control) and swatches not contaminated with bacteria and washed (transfer control). Swatches were challenged with both species simultaneously.

With clean gloved hands, the swatches (three technical replicates and transfer controls) were each attached to the edge of a single piece of standard polyester ballast using a safety pin. The safety pins were sterilized prior to usage by soaking in 70% ethanol and allowing for 30 min of contact time before the ethanol was decanted into a flask and the decontaminated pins placed in sterile Petri dishes to dry overnight. Each wash load was standardized to 2 ± 0.1 kg with standard ballast according to ISO 6330:2012 Textiles—Domestic washing and drying procedures for textile testing.^60^ This ballast accounts for washing with other items, providing added agitation, and working to prevent the swatches from floating on top of the water. The positive and negative controls were processed as soon as the washing cycle began.

### Washing of Swatches Using Different Detergents

The swatches were washed in a washing machine under three different conditions with varying temperatures and detergents that would feature in home laundering. A reference detergent, RD3, ISO 6330, was employed in each wash cycle. Additionally, the influence of bleach was studied.

In total, three types of detergent were used, (1) a nonbiological detergent, (2) a reference detergent (RD) 3 without optical brightener and enzymes (20 ± 1 g), and (3) RD3 with oxidative bleach (15.4 g RD3 + 4 g sodium perborate + 0.6 g TAED), which additive replaced 10% of the detergent. All detergents except the nonbiological detergent were purchased from SDC enterprise, Holmfirth, UK.

The washes took place using a domestic grade washing machine (Bush WMDF612 W 6KG 1200 Spin, Milton Keynes, UK). The machine has a set number of programs. Full programs were selected which had similar characteristics, including key temperature ranges. Two rinse cycles were desired; one program included two rinse cycles while another had only one, so an additional rinse cycle was added to the latter to enable comparisons. A temperature gauge (LogTag TRIX-8 data logger, New Jersey, USA) was used to monitor cycle settings. The gauge was designed to float in the wash, and measurement was by a temperature data logger. It was vacuum sealed to prevent water ingress and placed in a protective sleeve to prevent puncturing. Four washing cycles were tested: a 30°C rapid wash cycle for 15 min with a nonbiological detergent; a 40°C cycle for approximately 80 min with two rinse cycles (Easy care) using RD3 with TAED; a 40°C cycle for approximately 80 min with two 2 rinse cycles (Easy care) using RD3 without TAED and a 60°C cycle for 30 min (Daily 60 min) with a rinse cycle plus 1 manually added rinse cycle (36 min) using RD3 only. A laundering duration of 90 min was used to accommodate multiple rinse cycles, as per EN ISO 6330; in this case, there were two rinse cycles. Within each wash cycle, two placement conditions for the contaminated and transfer control swatches were studied. These conditions were as follows: washing with swatches secured in a wash bag and washing of swatches placed directly in the washing machine drum.

The temperature gauge was used during the washes. The washing machine was sterilized at the end of each experiment by running a 90°C wash program.

### Processing of Swatches for Bacterial Recovery and Enumeration

After washing, the swatches were aseptically removed with flame sterilized forceps into either a tumble dryer (Bush TD7CNBCW 7KG Condenser, Milton Keynes, UK) to dry for 30 min or under a class 2 safety cabinet to air-dry for 90 min. A total of 16 swatches were maintained in each wash cycle to accommodate the two drying methods, and each swatch was then aseptically removed using flame-sterilized forceps into a sterile stomacher bag. Ten mL of sterile PBS was added, and the sample processed in the stomacher for 4 min. At the end of the processing, PBS was removed (the swatch was manipulated to hang above the bottom of the stomacher bag and squeezed to release excess moisture of approximately 4 mL while within the sterile stomacher bag) and transferred to a sterile falcon tube. All samples were processed on the same day of the experiment. The positive control used for this study were bacteria contaminated swatches allowed to dry for 30 min under a class 2 safety cabinet and processed without washing. Preliminary work indicated that approximately 3 to 4 logs of test organisms were lost during air drying. Therefore, a high inoculum size was maintained to compensate for cell loss during drying. While humidity and temperature were not continually measured, a standard was maintained throughout the drying process by drying within a safety cabinet.

### Enumeration of the Cultivable Bacteria

All samples were analyzed by the following methods: Miles and Misra and filtration methods.

Fifty µl of each swatch PBS sample was removed after vigorous vortexing and serially diluted in sterile PBS from neat to 10^−^^3^ and processed using Miles and Misra counting technique on Cystine Lactose Electrolyte Deficient (CLED) agar (Oxoid, Basingstoke, UK). Agar plates were prepared at least 3 days prior and allowed to dry on the bench before usage to enhance easy drying of bacterial suspensions on the agar surface.

The remainder volume from each sample was filtered through a 0.45 µm PVDF membrane filter (Durapore, Cork, Ireland) with the aid of Merck Millipore-Sigma Microfil Filtration System 3 Head Vacuum Filter holder (Sigma Aldrich, Darmstadt, Germany). Filtration was facilitated with the aid of a high output vacuum pump, 220 V/50 Hz (Sigma Aldrich, Darmstadt, Germany). The filter was removed after filtration into a CLED plate using sterile forceps.

Plates were incubated overnight at 37°C, and the results read not more than 28 h after incubation started.

### Data Analysis

Characteristic colonies of test organisms recovered on membrane filters on CLED plates and from direct inoculation on CLED plates were counted and recorded after overnight incubation. All deviations in the recovered colonies were noted but were not considered in the hygienic efficacy of the laundering procedure. Only the test organisms were considered. Bacterial counts from filters or plates were reverted to average counts per swatch before the final values were converted to log values. All dilution factors were considered. Results were accessed on a fail or pass criterion based on >4 log reductions. Data obtained by Miles and Misra method were used only where colonies on filter membranes were too numerous to count.

## Results

Four different washing conditions were tested to determine the effectiveness of different detergent and temperature alternatives, as well as two drying alternatives, in the decontamination of three-layer swatches from a high-performing, cloth facepiece seeded with *P. aeruginosa* and *S. mutans*.

On average all 60°C and 40°C laundry cycles are highly effective with log reductions ranging from 3.96 to 7.04, primarily differing from the positive controls from which a recovery that ranged from log_10_5.09 to 7.04 CFU/ml was made. Although standards were maintained, there were variations in the positive controls across the different tests. Most of the artificially contaminated swatches were thoroughly cleaned, resulting in 0 CFU/swatch. At 40°C and 60°C, the number of cells recovered ranged from 0 to 50 CFU/swatch, with the highest count observed in swatches washed with RD fortified with oxidative bleach in a bag and dried in the air. Absolute values of CFU are well below the requirement for new medical masks and close to zero for both bacteria investigated.

The impact of detergent, temperature, and drying method was particularly evident at the lower temperature of 30°C. Swatches washed with nonbiological detergent at 30°C and air-dried had bacteria counts as high as 17,165 CFU/swatch (log_10_ 4.23). At 30°C efficient removal of bacteria was observed in combination with tumble drying for 30 min but not when air dried. In addition, leaving the swatches free in the drum during the 30°C wash cycle appears to be slightly more effective at bacteria removal than when they are contained within a wash bag.

Transfer rate of test isolates from artificially contaminated swatches to sterile transfer control swatches were highest with recoveries as high as 13 to 270 CFU/swatch when temperature of 30°C and nonbiological detergents plus air-dying were employed; this was followed by washing at 40°C with RD fortified with oxidative bleach. The transfer rate of test isolates from artificially contaminated swatches to sterile control swatches was high when washing at 40°C with RD fortified with oxidative bleach with numbers 13 and 38 CFU transferring to sterile control swatches. This was followed by washing at 30°C with nonbiological detergent followed by air drying, with recoveries ranging from 13 to 270 CFU/swatch.

The impact of temperature and detergent on the two test isolates, *P. aeruginosa* and *S. mutans*, was not significant. Both behaved similarly, with recovery occurring at all test temperatures of 30°C, 40°C, and 60°C (Table 1). Fewer bacteria were recovered at higher temperatures, with the highest numbers found at 30°C.

**Table 1.** Summary of Alternative Wash Conditions and Hygienic Efficacy of Each Laundering Procedure.

				CFU	CFU	Positive control		TUS (transfer)	Genus of bacteria
	Washed in	Dried	Replicate	Per swatch	Log/swatch	ln(10)	Log Red	CFU/swatch	Recovered
90 min wash	Drum	Air	T1	0	0.00	6.99	6.99	0	
60°C wash temperature			T2	13	1.10	6.99	5.89		*P*
2 Rinse cycles			T3	0	0.00	6.99	6.99		
Standard detergent	Bag	Air	T1	0	0.00	6.99	6.99	0	
Test 1			T2	3	0.40	6.99	6.59		S
			T3	0	0.00	6.99	6.99		
	Drum	Tumble	T1	0	0.00	6.99	6.99	0	
			T2	0	0.00	6.99	6.99		
			T3	0	0.00	6.99	6.99		
	Bag	Tumble	T1	0	0.00	6.99	6.99	0	
			T2	0	0.00	6.99	6.99		
			T3	0	0.00	6.99	6.99		
90 min wash	Drum	Air	T1	0	0.00	6.62	6.62	0	
60°C wash temperature			T2	0	0.00	6.62	6.62		
2 Rinse cycles			T3	0	0.00	6.62	6.62		
Standard detergent	Bag	Air	T1	0	0.00	6.62	6.62	0	
Test 2			T2	0	0.00	6.62	6.62		
			T3	0	0.00	6.62	6.62		
	Drum	Tumble	T1	0	0.00	6.62	6.62	0	
			T2	0	0.00	6.62	6.62		
			T3	0	0.00	6.62	6.62		
	Bag	Tumble	T1	0	0.00	6.62	6.62	5	*P, S*
			T2	0	0.00	6.62	6.62		
			T3	0	0.00	6.62	6.62		
90 min wash	Drum	Air	T1	20	1.30	7.04	5.74	0	*P, S*
60°C wash temperature			T2	0	0.00	7.04	7.04		
2 Rinse cycles			T3	0	0.00	7.04	7.04		
Standard detergent	Bag	Air	T1	0	0.00	7.04	7.04	0	
Test 3			T2	0	0.00	7.04	7.04		
			T3	0	0.00	7.04	7.04		
	Drum	Tumble	T1	8	0.88	7.04	6.16	0	*P*
			T2	0	0.00	7.04	7.04		
			T3	0	0.00	7.04	7.04		
	Bag	Tumble	T1	18	1.24	7.04	5.80	0	*P, S*
			T2	0	0.00	7.04	7.04		
			T3	0	0.00	7.04	7.04		
90 min wash	Drum	Air	T1	3	0.40	7.04	6.64	3	*S*
40°C wash temperature			T2	23	1.35	7.04	5.69		*P, S*
2 Rinse cycles			T3	0	0.00	7.04	7.04		
Standard detergent	Bag	Air	T1	0	0.00	7.04	7.04	0	
Test 1			T2	10	1.00	7.04	6.04		*P, S*
			T3	0	0.00	7.04	7.04		
	Drum	Tumble	T1	3	0.40	7.04	6.64		*S*
			T2	3	0.40	7.04	6.64		*S*
			T3	0	0.00	7.04	7.04		
	Bag	Tumble	T1	0	0.00	7.04	7.04	0	
			T2	0	0.00	7.04	7.04		
			T3	0	0.00	7.04	7.04		
90 min wash	Drum	Air	T1	0	0.00	6.35	6.35	5	
40°C wash temperature			T2	13	1.10	6.35	5.25		*P, S*
2 Rinse cycles			T3	15	1.18	6.35	5.17		*P, S*
Standard detergent	Bag	Air	T1	0	0.00	6.35	6.35	0	
Test 2			T2	18	1.24	6.35	5.11		*P, S*
			T3	3	0.40	6.35	5.95		*P*
	Drum	Tumble	T1	3	0.40	6.35	5.95	0	*S*
			T2	0	0.00	6.35	6.35		
			T3	0	0.00	6.35	6.35		
	Bag	Tumble	T1	3	0.40	6.35	5.95	0	*P, S*
			T2	0	0.00	6.35	6.35		
			T3	0	0.00	6.35	6.35		
90 min wash	Drum	Air	T1	0	0.00	4.96	4.96	0	
40°C wash temperature			T2	0	0.00	4.96	4.96		
2 Rinse cycles			T3	0	0.00	4.96	4.96		
Standard detergent	Bag	Air	T1	0	0.00	4.96	4.96	0	
Test 3			T2	3	0.40	4.96	4.56		*P*
			T3	0	0.00	4.96	4.96		
	Drum	Tumble	T1	10	1.00	4.96	3.96	0	*P, S*
			T2	3	0.40	4.96	4.56		*S*
			T3	0	0.00	4.96	4.96		
	Bag	Tumble	T1	5	0.70	4.96	4.26	0	*P, S*
			T2	3	0.40	4.96	4.56		*P*
			T3	3	0.40	4.96	4.56		*P*
90 min wash	Drum	Air	T1	0	0.00	6.47	6.47	0	
40°C wash temperature			T2	3	0.40	6.47	6.07		*P, S*
2 Rinse cycles			T3	0	0.00	6.47	6.47		
Standard detergent	Bag	Air	T1	0	0.00	6.47	6.47	13	*P, S*
Plus oxidative bleach			T2	3	0.40	6.47	6.07		*S*
Test 1			T3	0	0.00	6.47	6.47		
	Drum	Tumble	T1	10	1.00	6.47	5.47	38	*P, S*
			T2	18	1.24	6.47	5.23		*P, S*
			T3	15	1.18	6.47	5.29		*P, S*
	Bag	Tumble	T1	0	0.00	6.47	6.47	0	
			T2	0	0.00	6.47	6.47		
			T3	0	0.00	6.47	6.47		
90 min wash	Drum	Air	T1	0	0.00	6.89	6.89	0	
40°C wash temperature			T2	3	0.40	6.89	6.49		
2 Rinse cycles			T3	0	0.00	6.89	6.89		
Standard detergent	Bag	Air	T1	50	1.70	6.89	5.19	0	*P, S*
Plus oxidative bleach			T2	8	0.88	6.89	6.01		*P, S*
Test 2			T3	0	0.00	6.89	6.89		
	Drum	Tumble	T1	0	0.00	6.89	6.89	0	
			T2	0	0.00	6.89	6.89		
			T3	0	0.00	6.89	6.89		
	Bag	Tumble	T1	0	0.00	6.89	6.89	0	
			T2	0	0.00	6.89	6.89		
			T3	0	0.00	6.89	6.89		
90 min wash	Drum	Air	T1	0	0.00	5.47	5.47	0	
40°C wash temperature			T2	13	1.10	5.47	4.37		*P, S*
2 rinse cycles			T3	0	0.00	5.47	5.47		
Standard detergent	Bag	Air	T1	0	0.00	5.47	5.47	0	
Plus oxidative bleach			T2	3	0.40	5.47	5.07		
Test 3			T3	0	0.00	5.47	5.47		
	Drum	Tumble	T1	0	0.00	5.47	5.47	0	
			T2	0	0.00	5.47	5.47		
			T3	0	0.00	5.47	5.47		
	Bag	Tumble	T1	0	0.00	5.47	5.47	0	
			T2	0	0.00	5.47	5.47		
			T3	0	0.00	5.47	5.47		
15 min eco wash	Drum	Air	T1	2165	3.34	5.84	2.50	270	*P, S*
30°C wash temperature			T2	7335	3.87	5.84	1.97		*P, S*
1 Rinse cycles			T3	1835	3.26	5.84	2.58		*P, S*
Nonbio detergent	Bag	Air	T1	38350	4.58	5.84	1.26	148	*P, S*
Test 1			T2	1165	3.07	5.84	2.77		*P, S*
			T3	500	2.70	5.84	3.14		*P, S*
	Drum	Tumble	T1	3	0.40	5.84	5.44	0	*P*
			T2	0	0.00	5.84	5.84		
			T3	0	0.00	5.84	5.84		
	Bag	Tumble	T1	0	0.00	5.84	5.84	0	
			T2	0	0.00	5.84	5.84		
			T3	23	1.35	5.84	4.49		*P, S*
15 min eco wash	Drum	Air	T1	335	2.53	5.09	2.56	15	*P, S*
30°C wash temperature			T2	50	1.70	5.09	3.39		*P, S*
1 Rinse cycles			T3	58	1.76	5.09	3.33		*P, S*
Nonbio detergent	Bag	Air	T1	17000	4.23	5.09	0.86	13	*P, S*
Test 2			T2	500	2.70	5.09	2.39		*P, S*
			T3	36.675	1.56	5.09	3.53		*P, S*
	Drum	Tumble	T1	3	0.40	5.09	4.69	0	*P*
			T2	3	0.40	5.09	4.69		*P*
			T3	0	0.00	5.09	5.09		
	Bag	Tumble	T1	0	0.00	5.09	5.09	0	
			T2	3	0.40	5.09	4.69		*S*
			T3	3	0.40	5.09	4.69		*P,S*
15 min eco wash	Drum	Air	T1	5165	3.71	6.3	2.59	105	*P, S*
30°C wash temperature			T2	17165	4.23	6.3	2.07		*P, S*
1 Rinse cycles			T3	4000	3.60	6.3	2.70		*P, S*
Nonbio detergent	Bag	Air	T1	1335	3.13	6.3	3.17	158	*P, S*
Test 3			T2	9000	3.95	6.3	2.35		*P, S*
			T3	14000	4.15	6.3	2.15		*P, S*
	Drum	Tumble	T1	8	0.88	6.3	5.42	0	*P, S*
			T2	3	0.40	6.3	5.90		*P, S*
			T3	33	1.51	6.3	4.79		*P, S*
	Bag	Tumble	T1	0	0.00	6.3	6.30	0	
			T2	30	1.48	6.3	4.82		*P, S*
			T3	33	1.51	6.3	4.79		*P, S*

*+BS EN 14683: 2019—Microbial cleanliness < 30CFU/g.*

*Log reduction values are limited by the reduced value of the original dosing—positive control.

CFU: colony forming units, TUS: Transfer to Uncontaminated Swatches.

Washing with RD at temperatures 60°C and 40°C was effective in the removal of *P. aeruginosa* and *S. mutans* from swatches. Log 4 reduction was achieved, and transfer rates were minimal. Using nonbiological detergents at 30°C did not achieve the desired 4 log reduction and the transfer of test organisms between swatches were significant.

### Limitations to Result Analysis

As efficacy of each washing condition was determined by subtracting log number of bacteria recovered after wash from the log values obtained from positive control unwashed swatches, lower log reduction was seen where the positive controls were lower. This would appear as reduced efficacy of such washing conditions in comparison with wash batches with higher positive controls (Table 1).

Effective washing can prevent a clear view into subsequent processes, such as the effectiveness of drying. For procedures 1, 2, and 3, the wash cycle left the samples clean and well below the target 30 CFU/g, leaving insufficient bacteria to evaluate the drying process.

**Figure 1.**
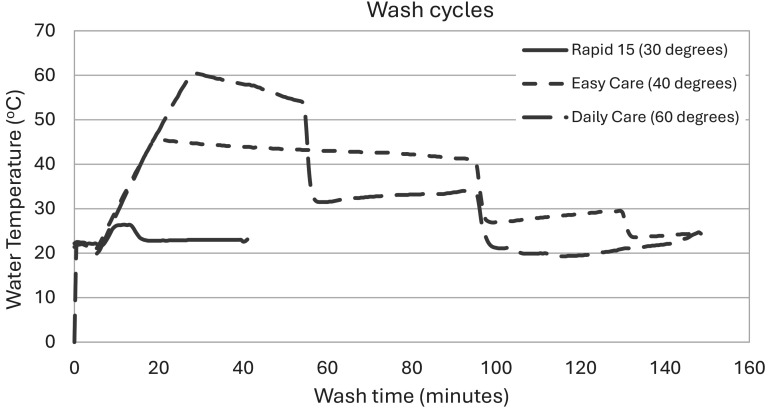
A chart showing the duration and temperature profile of each wash cycle as measured by a temperature probe. Rapid wash 15 (30°C) lasted for 40 min and the maximum temperature achieved as measured by a probe was 26.4°C, Easy care (40°C) lasted for 150 min with a maximum achieved temperature of 45.5°C, and Daily care (60°C) lasted for 150 min with a maximum achieved temperature of 60.5°C. The duration of the actual wash cycle differed from the time noted on the wash program.

It is noteworthy that in the cases where a temperature gauge was placed in the wash, the temperatures recorded were different from those specified in manufacturer settings. The actual wash times’ duration differed from the time duration on the machine program (Figure 1). Rapid wash 15 (30°C) lasted for 40 min, and the maximum temperature achieved as measured by a probe was 26.4°C, Easy care (40°C) lasted for 150 min with a maximum achieved temperature of 45.5°C, and Daily care (60°C) lasted for 150 min. The temperature of 30°C was not reached; instead, the maximum temperature fluctuated between 21.4°C and 24.1°C for 15 min. This outcome was reported here as <30°C since the target of 30°C was not achieved. The 40°C cycle setting fluctuated between 41°C and 45.4°C, and the 60°C setting fluctuated between 57°C and 60°C (Figure 1). Notably, this temperature range is only maintained for a relatively short part of the cycle. Such variations may be typical in domestic washing machines, and further research would be needed to determine their significance, if any.

## Discussion

The results of this study demonstrate a cleaning method that is effective in meeting an absolute benchmark performance level (adopted from BS EN 14683:2019) for a cloth facepiece whose functional performance after reprocessing is known to remain high.

This study evaluated laundering guidance provided for cloth facepieces intended for reuse and investigated whether reusable masks could be laundered under domestic laundering conditions in a safe and effective way at 60°C, 40°C, and <30°C. The results from laundering contaminated swatches demonstrate that standard washing machines and frequently used laundry techniques can clean a heavily contaminated face mask to the same standard of cleanliness as a new medical mask.

Results showed that all laundry cycles at 60°C and 40°C were highly effective, with absolute values of CFU close to zero, and that when using reference detergent 3, both washing temperatures met the cleanliness requirements for a new surgical mask, as such requirements are set forth in BS EN 14683:2019. The bacterial counts in this study are significantly higher than those typically found in contaminated facemasks, which range from 5 to 45.^51^ Using large inoculum sizes enables the reporting of log reductions.^61^

Where there is an oxidative bleach additive, the additive replaces 10% of the detergent which appeared to result in a slight decrease in performance (Table 1). Higher numbers of bacteria were recovered from the washed swatches compared to when only detergent was used. Additives like enzymes and bleach (chlorine and activated oxygen) are reported to be important for removing certain enteric viruses and bacteria at temperatures as low as 20°C. Bockmühl reported that oxidative bleach significantly reduced bio load at temperatures below 40°C.^62^ Although the efficacy of sodium perborate is described to be best at temperatures between 50°C and 60°C,^63^ its ability to enhance hygiene effectiveness in laundry, even at lower temperatures, was not seen in this study.

Results from this study reinforced that machine drying is another means to kill pathogens and showed that the effect is important when washing at low temperatures. High dryer temperatures can desiccate certain microbes and render them noninfectious. The results obtained in the study can be inferred to SARS-CoV-2 as many strains of coronavirus share common inactivation exposure temperatures of 60°C with bacteria species studied here.^64^ The SARS-CoV-2 virus has also been reported to be easily inactivated at 40°C.^65^ Such information is useful for managing this endemic virus in a community with access to mechanical dryers.

With respect to detergent, contamination transfer becomes significant when utilizing a lower wash temperature (30°C). When other factors such as detergent and wash temperature in a wash are less effective, washing the articles directly in a drum may be slightly more effective than washing in a bag. Generally, the present study found that contamination transfer within the wash may begin to occur when the effectiveness of the detergent is reduced; however, observed levels in the swatches tested still achieved the cleanliness requirements for new surgical masks.

**Key: Average CFU/swatch**: average number of CFU recovered across 3 swatches (technical replicates) in each of 3 biological replicates (3 swatches in each wash—wash repeated 3 times), **Max^+^ CFU/g**: Highest count recovered from any swatch/divided by weight of swatch (swatch weight = 4.7 g), Minimum log red*: Lowest log reduction for any swatch, Kills 99.99%:>Log 4 reduction achieved for all swatches reprocessed, **Max transfer**: largest count obtained from any transfer control swatch **Genus of bacteria recovered after laundry cycle:** Genus of bacteria recovered after laundry P = *Pseudomonas* S = *Streptococcus*.

The present study suggests a combination of factors can result in the hygienic reuse of cloth facepieces. To illustrate, bacteria were not inactivated to the benchmark level when swatches were washed at <30°C using nonbiological detergent in combination with air drying. In contrast, it was demonstrated that, with the addition of tumble drying, standard domestic laundry conditions at a temperature of less than 30°C using a nonbiological detergent can effectively clean heavily contaminated face masks to meet the same cleanliness standards as new surgical masks. A single approach is not proposed. When manufacturers research and develop care instructions for cloth facepieces, there is a need to distinguish among the approaches that result in the least bioburden from those that are not sufficient, depending on conditions. Performance levels must be based on common equipment and be in common use, for example, in relevant countries, to allow for easy adoption.

Effective reprocessing solutions can aid community adoption of reusable solutions and their sustainable use. The following are economic, environmental, and social considerations for reusable cloth facepieces and user instructions.

The economic benefits of reusable masks could be significant. Expert organizations such as American National Standards Institute (ASNI) and British Standards Institute (BSI) state a need for a more sustainable and robust supply chain to better meet current and future requirements. Higher value, long-lasting, reusable alternatives can be one way to achieve this outcome. There are valid concerns, however, that frequent washing of reusable facepieces could lead to material degradation and reduce protection,^66^ thereby affecting their economic viability. There is a market failure, however, when there is asymmetrical information (e.g., as to supply) relating to products that can meet an established demand. Recognizing the many practical considerations in manufacturing, marketing, and merchandizing facepieces, this investigation's studies evaluated facepiece performance before and after reprocessing. There are no consensus standards, however, which comprehensively detail methods for reprocessing cloth facepieces. Adjacent standards or guidance can help define suitable parameters and are available for consideration and assessment. For example, standards that might be used to guide medical textile reprocessing could be assessed for extension, with any needed qualifications, to facepieces of similar composition. AATCC has potentially relevant guidance covering testing for antimicrobial activity, shrinkage (dimensional change), colorfastness (which, as noted previously, can signal relative age to the user), and sustainability (release of fiber fragments in the laundering process). ASTM standards for tensile strength, tear resistance, seam strength, and abrasion resistance may be helpful in assessing possible degradation from reprocessing of facepieces with a similar composition.^67^

With respect to environmental sustainability, prior studies address how facepieces may affect the environment, including the benefits of reusable facepieces. A detailed enumeration of the environmental issues tied to reusability and reprocessing is beyond the scope of this study, yet a few issues have gained attention. Switching to reusable facepieces from disposable ones can cut greenhouse gas (GHG) emissions by 50% to 90%.^68,69^ Effective utilization and maintenance of reusable masks can lead to an 85% reduction in waste and result in a climate change impact that is 3.5 times lower, all while being 3.7 times more cost-effective^69^ . disposable facepieces, by design, are made for a single use with no reprocessing. Because they are intended for disposal after that use, their acquisition relates to the expected number of instances in which one needs to wear a facepiece. After use, discarding disposable facepieces can lead to solid waste issues when conducted in large volumes or at inappropriate locations; when use is at a lower-than-expected level, overstocking leads to potential waste. Given the solid waste issues, it would seem that reuse of disposable facepieces would provide environmental benefits. While studies suggest it is possible to achieve limited reuse of disposable facepieces, including respirators^70^ and medical masks,^71^ the nonwoven textiles used in filtering facepiece respirators (FFR), medical masks and similar facepieces typically degrade too easily for recurring use. Currently, manufacturer information and instructions, as well as US standards, do not address approaches to FFR reprocessing. In contrast, facepieces which, by design, can be reprocessed then reused, would have a longer product life, reinforcing the need for accepted methods for demonstrating that they remain effective after reprocessing.

From a social sustainability perspective, the focus is on the equities of the wearer and the community, including vulnerable or special populations. There are health equity issues that require clearer guidance. If not adequately cleaned and maintained, reusable facepieces could pose risks from the accumulation of microorganisms or toxic materials. Further, home laundering as an infection control measure presents risks at each step in the process.^72^ However, unlike environmental considerations aided by government and legal processes, there are few entry points for communicating social equities to manufacturers during the design process. Collaborative efforts at assisting the community are necessary, and some are in place, pursuing approaches to important technical issues. At a minimum, manufacturer information and instructions must provide details on inspection, care, and other tasks that a wearer must understand and perform to mitigate risk from the use of facepieces designed to be reusable and reprocessed.

Future research could examine relevant factors in more detail. Such research might consider the real or perceived risks from use of reusable cloth facepieces in the community, as they relate to the following:

- Limited understanding of microbial and other hazards to which a cloth facepiece may be exposed in a community setting having no infection control program.
- Limited evidence and a lack of consensus on models and surrogates for testing the reprocessing of cloth facepieces exposed to hazards in a community setting.
- For facepieces designed for repeated reuse and reprocessing, it is important to expand or revise the suite of relevant test methods. Testing of materials alone for filtration and breathing resistance performance, for example, may be useful for disposable facepieces which are not expected to be subjected to reprocessing and, thereby, possible degradation; however, whole product testing is important to assessing a reusable facepiece's performance over time.
- Lack of standards or other means for managing the influence of introduced interventions and confounding factors, including machine washing (impacts on filter, breathability, seal, and overall protective performance from, e.g., detergent, agitation, wear on harness, wear on filter), dryer use, product aging, hand hygiene, handling during use including repeated donning and doffing.

There were several limitations in this research which may prevent its generalization to all settings where cloth facepieces are used in the community. With respect to the surrogates tested, only two were used, and while they were selected for their alignment to relevant aspects of respiratory disease transmission, other pathogens might be more difficult to inactivate or remove. A test for the inactivation of a bioaerosol was chosen, given relevance to infectious diseases transmission and the human biome. However, this study did not examine the removal of hazards, for example, fungal spores, that could survive washing conditions. Not addressed in the test method were potentially toxic chemicals or inorganic or inert particles, for example, nuisance dust, which may adhere to a cloth face mask and require reprocessing.

With respect to study limitations in reproducing at-home laundering conditions, under real conditions there will be variability in effectiveness for the protocols used, by machine type and agitation method, for example. With respect to the range of wash programs evaluated, it is noteworthy that society often is encouraged to carry out quicker, more economic (“eco”) wash cycles which save on energy costs; it may be beneficial to conduct more detailed examination of which options might be effective and under what conditions.

The washing machine model manual did not provide information on the water volume used per cycle, making it difficult to determine if there were variations in volume between cycles or assess their potential impacts. Another limitation considered is that the dryer was not sterilized in between runs, unlike the washing machine which was sterilized at end of each of experiment by running a 90°C wash program.

With regard to the laundry cycles for the performance testing, the subject facepiece was not worn, subjected to artificial aging, or challenged with a surrogate pathogen or other contaminant.

In conclusion, society, including manufacturers, should consider sustainable alternatives to disposable facepieces. At the same time, public health and safety requires evidence for such alternatives. This research contributes to an understanding of how domestic laundering can maintain facepiece hygiene through validation of a proposed performance benchmark for cleanliness. Results support sustainable design, testing, and manufacture of facepieces and enable their unassisted reprocessing and reuse to manage respiratory hazards in the community.

## Supplemental Material

sj-docx-1-new-10.1177_10482911251334843 - Supplemental material for Demonstration of a Home Laundering Method for Cloth Facepieces to Achieve Hygienic and Sustainable Reuse Supplemental material, sj-docx-1-new-10.1177_10482911251334843 for Demonstration of a Home Laundering Method for Cloth Facepieces to Achieve Hygienic and Sustainable Reuse by William Mackay, Chris Baglin, Paul Baglin, Claire Chalmers, Fiona Henriquez and Ngozi Amaeze in NEW SOLUTIONS: A Journal of Environmental and Occupational Health Policy

sj-docx-2-new-10.1177_10482911251334843 - Supplemental material for Demonstration of a Home Laundering Method for Cloth Facepieces to Achieve Hygienic and Sustainable Reuse Supplemental material, sj-docx-2-new-10.1177_10482911251334843 for Demonstration of a Home Laundering Method for Cloth Facepieces to Achieve Hygienic and Sustainable Reuse by William Mackay, Chris Baglin, Paul Baglin, Claire Chalmers, Fiona Henriquez and Ngozi Amaeze in NEW SOLUTIONS: A Journal of Environmental and Occupational Health Policy

sj-docx-3-new-10.1177_10482911251334843 - Supplemental material for Demonstration of a Home Laundering Method for Cloth Facepieces to Achieve Hygienic and Sustainable Reuse Supplemental material, sj-docx-3-new-10.1177_10482911251334843 for Demonstration of a Home Laundering Method for Cloth Facepieces to Achieve Hygienic and Sustainable Reuse by William Mackay, Chris Baglin, Paul Baglin, Claire Chalmers, Fiona Henriquez and Ngozi Amaeze in NEW SOLUTIONS: A Journal of Environmental and Occupational Health Policy

sj-docx-4-new-10.1177_10482911251334843 - Supplemental material for Demonstration of a Home Laundering Method for Cloth Facepieces to Achieve Hygienic and Sustainable Reuse Supplemental material, sj-docx-4-new-10.1177_10482911251334843 for Demonstration of a Home Laundering Method for Cloth Facepieces to Achieve Hygienic and Sustainable Reuse by William Mackay, Chris Baglin, Paul Baglin, Claire Chalmers, Fiona Henriquez and Ngozi Amaeze in NEW SOLUTIONS: A Journal of Environmental and Occupational Health Policy

sj-docx-5-new-10.1177_10482911251334843 - Supplemental material for Demonstration of a Home Laundering Method for Cloth Facepieces to Achieve Hygienic and Sustainable Reuse Supplemental material, sj-docx-5-new-10.1177_10482911251334843 for Demonstration of a Home Laundering Method for Cloth Facepieces to Achieve Hygienic and Sustainable Reuse by William Mackay, Chris Baglin, Paul Baglin, Claire Chalmers, Fiona Henriquez and Ngozi Amaeze in NEW SOLUTIONS: A Journal of Environmental and Occupational Health Policy

sj-docx-6-new-10.1177_10482911251334843 - Supplemental material for Demonstration of a Home Laundering Method for Cloth Facepieces to Achieve Hygienic and Sustainable Reuse Supplemental material, sj-docx-6-new-10.1177_10482911251334843 for Demonstration of a Home Laundering Method for Cloth Facepieces to Achieve Hygienic and Sustainable Reuse by William Mackay, Chris Baglin, Paul Baglin, Claire Chalmers, Fiona Henriquez and Ngozi Amaeze in NEW SOLUTIONS: A Journal of Environmental and Occupational Health Policy

sj-docx-7-new-10.1177_10482911251334843 - Supplemental material for Demonstration of a Home Laundering Method for Cloth Facepieces to Achieve Hygienic and Sustainable Reuse Supplemental material, sj-docx-7-new-10.1177_10482911251334843 for Demonstration of a Home Laundering Method for Cloth Facepieces to Achieve Hygienic and Sustainable Reuse by William Mackay, Chris Baglin, Paul Baglin, Claire Chalmers, Fiona Henriquez and Ngozi Amaeze in NEW SOLUTIONS: A Journal of Environmental and Occupational Health Policy

sj-docx-8-new-10.1177_10482911251334843 - Supplemental material for Demonstration of a Home Laundering Method for Cloth Facepieces to Achieve Hygienic and Sustainable Reuse Supplemental material, sj-docx-8-new-10.1177_10482911251334843 for Demonstration of a Home Laundering Method for Cloth Facepieces to Achieve Hygienic and Sustainable Reuse by William Mackay, Chris Baglin, Paul Baglin, Claire Chalmers, Fiona Henriquez and Ngozi Amaeze in NEW SOLUTIONS: A Journal of Environmental and Occupational Health Policy

sj-docx-9-new-10.1177_10482911251334843 - Supplemental material for Demonstration of a Home Laundering Method for Cloth Facepieces to Achieve Hygienic and Sustainable Reuse Supplemental material, sj-docx-9-new-10.1177_10482911251334843 for Demonstration of a Home Laundering Method for Cloth Facepieces to Achieve Hygienic and Sustainable Reuse by William Mackay, Chris Baglin, Paul Baglin, Claire Chalmers, Fiona Henriquez and Ngozi Amaeze in NEW SOLUTIONS: A Journal of Environmental and Occupational Health Policy
