# Supplementary material for: Demonstration of a Home Laundering Method for Cloth Facepieces to Achieve Hygienic and Sustainable Reuse
Source: New Solut. 2025 May 8;35(2):173–87. doi: 10.1177/10482911251334843 (PMC12222837; doi:10.1177/10482911251334843)
Supplement: sj-docx-1-new-10.1177_10482911251334843 - Supplemental material for Demonstration of a Home Laundering Method for Cloth Facepieces to Achieve Hygienic and Sustainable Reuse [file sj-docx-1-new-10.1177_10482911251334843.docx]

**Supplement**

*Standards of interest to reusable facepiece manufacturers and users*

ASTM D3938, Standard Guide for Determining or Confirming Care Instructions for Apparel and Other Textile Products, section 8.5, calls for the selection of test methods for evaluation of the functional properties of a given textile product after its reprocessing, while its section 8.6 calls for selection of a performance level^1^. ASTM F3502 Standard Specification for Barrier Face Coverings provides test methods which are applicable to reusable and cloth facepieces. F3502 requires performance testing for filtration and breathing resistance, in its condition at first use and after the number of reprocessing sessions identified for its lifespan. Its approach to validating reusability relies solely on accelerated aging via repeated reprocessing, rather than testing with simulated wearing before reprocessing. With respect to fit F3502 specifies an optional “leakage assessment” to help manufacturers address fit performance before and after reprocessing. Drawing from ASTM F3502’s leakage assessment method, NIOSH developed performance levels for fit, Enhanced Performance and Enhanced Performance Plus, which a manufacturer can apply to a cloth facepiece after a given number of reprocessing intervals and thereby assess its quality.

AATCC M14-2020, Guidance and Considerations for General Purpose Textile Face Coverings: Adult, states that hand laundering should be avoided and each laundering cycle should include washing and drying procedures^2^. This guidance also provides that, when determining care, consideration should be given to the most sensitive element, such as the textile which is most prone to dimensional change. Intervals for testing performance are selected and, at each interval, an evaluation is carried out on a different area of the filter and the assembly, as well as the attachment and elastic recovery of loops/ties, using ASTM D4964 and ASTM D5034. A fuller evaluation protocol is in AATCC M14-2020 section 17.1 and its Table 1. AATCC M-14-2020 Table 1 outlines laundering and care considerations, recommending in part that there must be instructions for home laundering and information on service life that describes the maximum number of laundering cycles. As noted previously, in its Table 1, AATCC M14-2020 also provides guidance on the selection of fabric types whose known properties make them candidates for use in cloth face coverings which may withstand the general laundering approach in the guidance. Its section 4.11 lists several properties which define service life: user fit, particle filtration, breathing resistance, ear loop or tie attachment, and ear loop or tie elastic recovery (if applicable). These properties are potential performance areas for measurement and *reassessment after reprocessing, for consideration in standards development.*

*Prior studies of laundering conditions*

In textile washing studies, the temperature levels assessed largely range from 30 ^o^C to 80 ^o^C, as reported by Linke and colleagues^3^. Hansen observed that washing at a 60 ^o^C temperature for 30 minutes (followed by drying in open air) did not reduce pathogens to desired levels ^4^ . In another context, under a different set of variables, additives such as enzymes and bleach (chlorine and activated oxygen) were important for the removal of certain enteric viruses and bacteria at temperatures as low as 20^o^C^5^. Bockmühl also reported that oxidative bleach significantly reduced biological load at temperature below 40 ^o^C^6^ in further contrast to the 60 ^o^C temperature common to Table S1.

The standard EN ISO 6330, which outlines domestic washing and drying procedures for textile testing, requires multiple rinse cycles, including up to four. Multiple cycles can help ensure the washing is effective for fabrics with known properties, such as a likelihood of binding with microbes. Longer wash cycles are less convenient for the domestic operator but do not necessarily consume more energy when the added time is soaking time.

Washing in combination with tumble drying provides effective reprocessing. Tano and Melhus tested “hospital textiles” at Sweden’s national 70 ^o^C temperature alongside 60 ^o^C^7^. These investigators found that washing reduced the number of bacteria with 3-5 log_10_ CFU, and subsequent tumble drying reduced the bacterial numbers another 3-4 log_10_ CFU. They noted that without tumble drying, bacterial risk remained from the 60 ^o^C wash and had originated from the washing cycle. Another group of authors reviewing relevant studies noted that some investigators found machine drying to be the most effective form of decontamination^8^.

*Sampling of washing guidance for reusable cloth masks*

Washing temperature is a key aspect of laundry practices. Table S1 lists sources of guidance for reprocessing reusable cloth face coverings.

**Table S1. Sampling of guidance for the laundering of cloth face coverings intended for reuse as an intervention in the transmission of respiratory diseases.**

| Organization | Guidance | Washing | Drying |
| --- | --- | --- | --- |
| AATCC (American Association of Textile Chemists and Colorists) | Guidance and Considerations for General Purpose Textile Face Coverings: Adult 9 ^2^ | Follow all applicable local and national regulations. | Follow all applicable local and national regulations. |
| AFNOR (French: Association Française de Normalization; English: French Standardization Association) | Barrier masks Guide to minimum requirements, methods of testing, making and use ^9^ | 60°C for 30 minutes |  |
| ANSM (French: Agence nationale de securite du medicament et des produits de sante; English: French National Agency for Medicines and Health Products Safety) | Specifying the treatment protocol [allowing reuse of fabric masks for non-sanitary use provided for in the context of the covid epidemic ^10^ | At home, machine-wash with a standard detergent at 40 degrees, 30 minutes. In commercial laundries, 30 minutes at 60 °C | Machine or air dry |
| ASTM International | F3502 Standard Specification for Barrier Face Coverings ^11^ | As specified by manufacturer. | As specified by manufacturer. |
| BSI (British Standards Institute) | BSI Flex Version 2.1 - Community face coverings – Specification ^12^ | At home: Minimum of 40°C using a normal wash procedure.  Communal laundry facility: 70 °C minimum (as defined by  BS EN ISO 15797) |  |
| CDC (US Centers for Disease Control and Prevention) | Use and Care of Masks ^13^ | Wash by hand or use a washer. | Dry by hand or use a dryer. |
| CEN (European Committee for Standardization) | CEN CWA 17553: 2022 Community face coverings - Guide to minimum  requirements, methods of testing and use (June 2020) ^14^ | 40°C minimum with the use of consumer laundry detergent. 60°C if the use of consumer laundry detergent is not instructed. Duration of washing machine cycle of minimum of 30 minutes (normal settings – not the economic settings) | Follow either the claimed detailed cleaning instructions or the procedure described in EN ISO 6330. |
| GINETEX (International Association for Textile Label Care) | HOW TO CLEAN A FABRIC FACE MASK IN THE TIME OF COVID-19^15^ | 60°C for 30 minutes with a classic laundry soap | 60°C minimum in a dryer (delicate textile).  Natural drying in a clean and ventilated area. |
|  |  |  |  |
| National Health Commission (People’s Republic of China) | Prevention and control program of COVID-19 (6th edition) ^16^ | Heating for a time period of 30 minutes at 56°C. |  |
| WHO (World Health Organization)/ | Infection prevention and control in the context of coronavirus disease (COVID-19): A living guideline 2022 Apr 25 ^17^ | At least 60°C /140°F with soap or detergent  If <60°C/140°F, with soap or detergent followed by boiling for 1 minute |  |

*Impacts of repeated wash cycles on filtration, breathing resistance, and fit.*

By design, the facepiece used in this study’s experiments incorporated robust, traditionally constructed fabrics to ensure its structure is not easily damaged or degraded by laundering. The facepiece has been manufactured with either of two filter types (designated A (warp-knitted polyester fleece) or B (non-woven polypropylene)). While only Filter A was used in the present study, it is helpful to review both. For each filter, the middle of 3 layers was designed to filter smaller particles, while the outer layers filter large particles, protect the inner filter, and provide moisture control. The outside layer is warp-knitted polyester and the inside layer is warp-knitted nylon). Further, the construction of the facepiece is such that consistent functionality and performance are not reliant on retaining its original shape after washing.

For Filter A, a knitted material, the filtration performance requirement was set at 3um in line with BS EN 14683 for medical face masks and CWA17553 2020 for Community Face Coverings. For Filter B, a non-woven material, the filter performance requirement was set at 0.3um in line with ASTM F3502 for Barrier Face Coverings.

With respect to filtration and breathing resistance testing, test methods and performance targets were the BFE filtration requirement from BS EN 14683:2019 Medical Face Masks and those provided in ASTM F3502 Barrier Face Coverings^11^ as they pertain to testing of the entire facepiece. For the purposes of this paper, there was a reliance on results from filtration and breathing resistance performance testing by independent test laboratories that had been reported previously in Baglin et al. That paper reported on the same two filter types, Filter A and Filter B. As noted, Filter A was the filter embedded in the samples used for the wash samples for this study.

With respect to fit performance, two methods were used to assess the facegaiter. The set of facegaiters with Filter A were tested in-house repeatedly for Total Inward Leakage using an in-house method on a manikin over 200 wash cycles. Facegaiters with Filter B were used to perform Quantitative Leakage Assessment testing on human subjects using the optional quantitative test method from ASTM F3502 together with the target performance levels specified by the Centers for Disease Control and Prevention’s (CDC) Enhanced Performance/Enhanced Performance Plus (formerly known as Workplace Performance/Workplace Performance Plus) framework^18^. In this framework, CDC employs the ASTM F3502 quantitative leakage assessment test method to enable an assessment of performance as fit^19^. CDC described this framework on a public webpage^20^, and it lists separately those facepieces conforming to its performance levels for fit, explaining, “Leakage is reported as a ratio of the particle concentration outside the face covering over particle concentration inside the face covering^21^. Higher leakage ratios indicate less leakage.” The method uses a TSI PortaCount CNC particle counter in the N99 mode, which includes particles that could penetrate the filter and, therefore, measures the performance of the entire facepiece as worn i.e. leakage through the filter (filtration) and leakage around the edges of the facepiece (fit).

*Results for Filter A*

Regarding filter performance after washing, Table S2 presents data from independent testing^22^ of BS EN 14683 Medical face masks. This test assesses the survivability of *Staphylococcus aureus* using a wet aerosol and cascade impactor with a size range 0.65 μm – 7.0 μm, mean particle 3.0 um, and velocity 9.6 cm/s. BFE filtration showed filtration levels >99% after 100 laundry cycles, both on and off-seam (the stitching that holds the 3 fabric layers together). The pass mark at performance level II is >98%^22^.

**Table S2 Results of independent testing to EN14683 mean particle size 3um.**


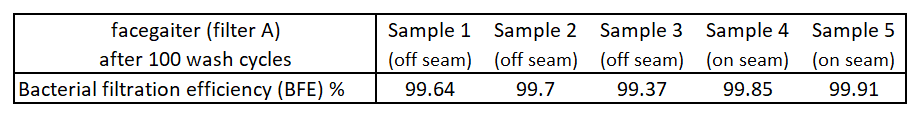


Regarding breathing resistance requirements after washing, independent flow resistance measurements to ASTM F3502 were carried out for 10 samples after 1 wash and after 100 wash cycles. The airflow resistance increases from an average of 1.32mm to an average of 1.62mm. Although the flow resistance increases by around 25% after repeated wash cycles this flow resistance is still very low both before and after laundering, the pass mark at the highest performance level is <5mm (Table S3), and typical values for respirators are about 10mm.

**Table S3. Airflow Resistance of a size medium facegaiter, tested over the full area of the filter at 85Lpm after 1 and 100 laundry cycles**


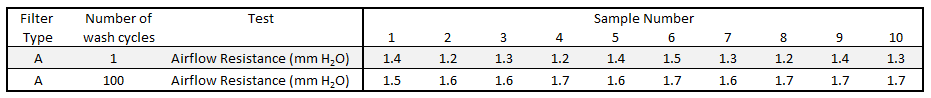


The effective area of the test sample is 372cm^2^. For fit performance after washing, in-house testing of the Filter A facegaiter as worn on a static manikin under tidal breathing at 30 l/min (peak flow 94 l/min) was carried out repeatedly over 200 wash cycles (Figure S1). Six samples were laundered at 40^o^C and six samples at 60^o^C. The test measures the Total Inward Leakage including edge and filter penetration to indicate the combined effect of the filter, harness and seal on the overall facepiece performance. The challenge was dried NaCl polydisperse aerosol. Dual sampling was carried out using a 5 channel OPC across the range of 0.3um to 10um. The samples were not pre-conditioned prior to testing and the aerosol was not neutralized. The results show a substantial improvement in the performance after the first wash cycle, which is presumed to reflect laundering’s removal of any finishing treatments from the fabric manufacturing process. The overall general trend shows a 2% reduction in performance over 200 wash cycles for both the 40 ^o^C and 60^o^C degree samples. The difference in average performance between the two wash temperatures is less than 1%.

*Fig S1 Overall facepiece performance (with Filter A) by aerosol mass across the range 0.3um - 10um*

The results for the 60^o^C cycles are shown below for the two particle size ranges most closely associated with the CWA17553 and ASTM F3502 test methods, 2-5 um and 0.3-0.5 um respectively (Figure S2).

*Figure S2 Overall facepiece performance (with Filter A) after washing at 60^o^C – for specific particle size bins (pre-wash values removed).*

ASTM F3502 Barrier Face Coverings calls for filter performance to be tested using the method described in US regulation 42 CFR Part 84.170, the test method used for N95 respirators^23^. ASTM F3502 specifies a different minimum filtration performance for level II (at 50%), in contrast to 95% for an N95. Under this method, the average particle size by mass is 0.27um and the average by count is 0.075um. Based on observations, the filtration of particles towards the bottom end of this size range can be unpredictable for Filter A and can cause a larger variation in results for that filter making a consistent measurement of performance difficult; Filter A was designed to remove 3.0um particles. Therefore, to demonstrate that a washable filter can also perform at or close to the requirements for a respirator, another filter arrangement, Filter B, was evaluated.

*Results for Filter B*

The filter performance and breathing resistance of the facegaiter with Filter B were independently tested to ASTM 3502 after 1 and 50, wash cycles at 60^o^C. The preconditioned samples were tested as a complete facepiece (size medium) clamped into an adapter plate and placed directly into a TSI 8130A filter tester. The aerosol is dried and neutralized NaCl with a mean particle size of 0.27um. The flow rate is 85 l/min and the area inside the clamp, less obstructions caused by supports, is 372cm^2^.

The independent laboratory results for breathing resistance are reproduced in Table S4. Airflow resistance increases from an average of 3.86mm to an average of 4.47mm after 50 wash cycles i.e., an improved filter performance. The performance for the filter is slightly below the requirements for an N95 after 1 wash and comfortably above the requirements after 50 washes. For the purpose of ASTM F3502, the lowest performance is adopted as the product performance level.

**Table S4. Airflow Resistance of a size medium facepiece (with Filter B), tested over the full area of the filter at 85Lpm after 1 and 50 laundry cycles**


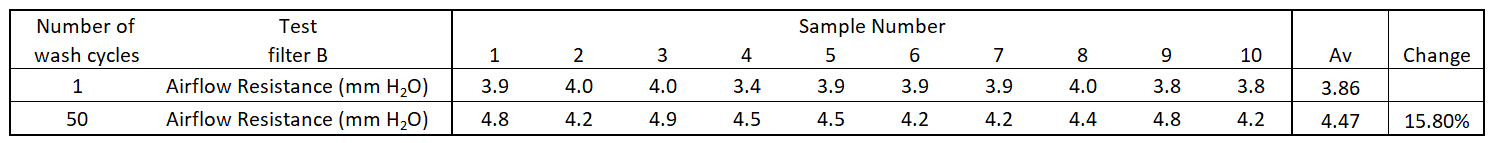


This change corresponds to a 15% increase in the effort required to force air (breath) through the filter. However, the value is still well below the maximum allowable limit of 15mm and still below the preferred upper limit of 5mm, in ASTM F3502 and the limits allowed for respirators.

Table S5 reproduces the independent laboratory results for filtration. After 50 wash cycles, filter performance increases from an average of 95.19% to an average of 97.79%. This change corresponds to a reduction in filter penetration of just over 50%. The filter's performance is slightly below the requirements for an N95 after 1 wash and comfortably above the requirements after 50 washes.

**Table S5. Filtration performance of a size medium facepiece (with Filter B), tested over the full area of the filter at 85Lpm after 1 and 50 laundry cycles**


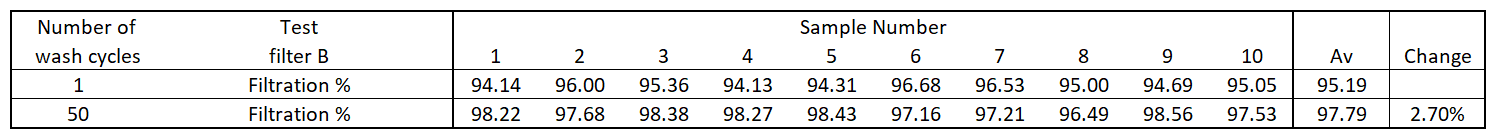


The fit of the facegaiter with Filter B was also assessed after a minimum of 110 wash cycles at 60^o^C. A minimum is referred to because, consistent with Ethics approvals, some masks were washed and used more than once with different human subjects. The method used was the optional, Quantitative Leakage Assessment test method in ASTM F3502. The ASTM F3502 test method measures leakage when worn by a subject and determines the Total Inward Leakage (using the N99 mode of the TSI PortaCount 8048) of particles in the .02 um to 1um range by the count method (each particle counts as 1, irrespective of size and because the smaller particles are more numerous the weighting will be towards the lower end of the size range). Results were submitted to CDC’s NIOSH and approved for listing as a Barrier Face Covering under the “Enhanced Performance Plus” performance level.

Table S6 presents the fit performance of this reusable facepiece, which exceeded the CDC’s Enhanced Performance Plus performance target, using the ASTM F3502 test method; and fit performance improved over the course of repeated wash cycles. Leakage ratios increased substantially after repeated wash cycles (indicating better performance). When expressed in terms of percent leakage, the results show that the average Total Inward Leakage reduced from 6.8% after the 1^st^ wash to 4.4% after 110 wash cycles, indicating more particle-blocking capability over time.

**Table S6. Summary of Leakage Ratios of the facepiece (with Filter B).**


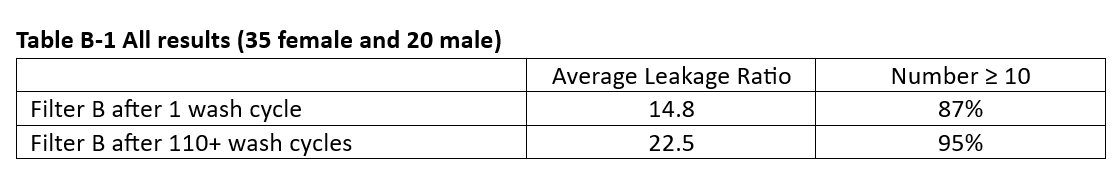


**The passmark for NIOSH’s Enhanced Performance Plus level is the average leakage ratio of at least 10 subjects. The column indicating a Leakage Ratio of “Number ≥ 10” gives the percentage of test subjects achieving or exceeding this pass mark.*

*Results Summary*

With respect to filter performance after washing, Filter A met the requirement of a Type II Medical Face Mask with >99% filtration at 3.0um after 100 wash cycles. The filtration performance of Filter B, when tested to ASTM F3502 at 0.3um, increased after 50 wash cycles, with the filter penetration reducing by around 50%, from 6% to less than 3% (>97% filtration).

With respect to breathing resistance after extensive wash cycles, the flow resistance of both filters increased slightly, however, both filters maintained a flow resistance of <5mm H_2_O, the higher performance level under ASTM F3502, and much less (equating to greater comfort) than the requirement for respirators.

With respect to fit, for Filter A in-house Total Inward Leakage tests on a manikin show a combined drop in performance (fit and filtration) of around 2% over the course of 200 wash cycles while still achieving the target performance for both 40^o^C and 60^o^c wash cycles. For Filter B the performance targets were met for subjects wearing full cloth facepieces after 1 wash cycle and after 110 wash cycles, with the overall performance increasing as shown in Table S6, which summarizes relevant data from a separate study. CDC reviewed the study’s fit test results from 55 subjects, assessed it for alignment with Enhanced Performance/Performance Plus, and accepted it as evidence of conformity in a public list online^24^.

Results indicate that extensive reprocessing, in many cases more than 100 wash cycles, did not reduce the levels of performance below those required by the relevant standards. In some cases, the performance after extensive washing exceeded that after a single wash.

For all results reported in this Supplement all laundering cycles include machine drying (tumble drying) on a low heat setting. Independent testing to relevant standards involved preconditioning of samples prior to testing.

The foregoing describes test methods similar to, but different from, those for a respirator. The testing presented does not meet the requirements of 42 CFR Part 84 for an N95 respirator.

**References**

1. ASTM. D3938-18: Standard Guide for Determining or Confirming Care Instructions for Apparel and Other Textile Products., <https://www.astm.org/d3938-18.html> (2018).

2. AATCC. M14 Guidance and Considerations for General Purpose Textile Face Coverings: Adult., <https://members.aatcc.org/store/m014/3085/> (2020, accessed 4th January 2023).

3. Linke S, Gemein S, Koch S, et al. Orientating investigation of the inactivation of Staphylococcus aureus in the laundry process. *Hyg Medizin* 2011; 36: 8-12.

4. Hansen J-C, Planchette G, Cavedon J-M and Julien H. Contribution à l’étude de l’efficacité et de l’entretien des masques de protection respiratoire COVID en tissu fait maison. *Médecine de Catastrophe-Urgences Collectives* 2021; 5: 308-316.

5. Abney SE, Ijaz MK, McKinney J and Gerba CP. Laundry hygiene and odor control: state of the science. *Applied and environmental microbiology* 2021; 87: e03002-03020.

6. See reference 64 in the main document.

7. See reference 46 in the main document.

8. Kagan LJ, Aiello AE and Larson E. The role of the home environment in the transmission of infectious diseases. *Journal of community health* 2002; 27: 247-267.

9. See reference 58 in the main document.

10. ANSM. AVIS du 25 mars 2020 revise le 02/11/2020 V4 Precisant le protocole de traitement permettant une reutilisation des masques en tissu a usage non sanitaire prevus dans le cadre de l'epidemie COVID Saint Denis, Fr, <https://www.entreprises.gouv.fr/files/files/enjeux/covid-19/20201127_avis_ansm_protocole_nettoyage_masquesv4-1.pdf> (2020, accessed 4th January 2023).

11. ASTM A. F3502-21 standard specification for barrier face coverings. *ASTM International: West Conshohocken, PA, USA* 2021.

12. BSI. BSI Flex Version 2.1 Community face coverings — Specification. London, UK., <https://www.bsigroup.com/en-GB/topics/novel-coronavirus-covid-19/community-face-coverings/> (2021, accessed 4th January 2023).

13. CDC. Use and Care of Masks. Atlanta, GA, USA, <https://www.cdc.gov/coronavirus/2019-ncov/prevent-getting-sick/about-face-coverings.html> (2022, accessed 4 September 2020 2023).

14. ECS. CEN CWA 17553: 2022. Community face coverings - Guide to minimum requirements, methods of testing and use. Brussels, Belgium.2022, <https://standards.cencenelec.eu/dyn/www/f?p=CEN:110:0::::FSP_PROJECT,FSP_ORG_ID:73484,6229&cs=16F7D93ABF74ABC2B6CD328D5BDBF7BEC> (2022, accessed 4th January 2023).

15. Ginetex. The International Association for Textile Care and Labelling. How to clean a fabric face mask in the time of covid-19. 2022, <https://www.ginetex.net/article/GB/how-to-clean-a-fabric-face-mask-in-the-time-of-covid-19> (2023, accessed 4th January 2023).

16. NHCPRC. Protocol for Prevention and Control of COVID-19 (Edition 6). *China CDC Wkly* 2020; 2: 321-326. DOI: 10.46234/ccdcw2020.082.

17. WHO. Infection prevention and control in the context of coronavirus disease (COVID-19): a living guideline, 13 January 2023. *Infection prevention and control in the context of coronavirus disease (COVID-19): a living guideline, 13 January 2023*. 2023.

18. Brosseau LM and Stull J. Criticism of workplace protection barrier face covering article mischaracterizes ASTM standard and its potential utility. *NEW SOLUTIONS: A Journal of Environmental and Occupational Health Policy* 2023: 10482911231211319.

19. Brosseau LM and Stull J. Barrier face coverings for workers. *NEW SOLUTIONS: A Journal of Environmental and Occupational Health Policy* 2022; 32: 182-188.

20. CDC. Making Enhanced Performance Barrier Face Coverings, <https://www.cdc.gov/niosh/topics/publicppe/barrier-face-coverings.html> (2023, accessed 4 February 2024).

21. CDC. Barrier Face Coverings and Enhanced Performance/Performance Plus Masks, Conforming BFCs and Enhanced Performance/Performance Plus Masks, <https://wwwn.cdc.gov/PPEInfo/RG/FaceCoverings> (2023, accessed 4 February 2024).

22. See reference 40 in the main document.

23. See reference 93 in the main document.

24. eCFR. 42 CFR Part 84 Subpart K Air-Purifying Particulate Respirators. 2002.
