# Supplementary material for: Demonstration of a Home Laundering Method for Cloth Facepieces to Achieve Hygienic and Sustainable Reuse
Source: New Solut. 2025 May 8;35(2):173–87. doi: 10.1177/10482911251334843 (PMC12222837; doi:10.1177/10482911251334843)
Supplement: sj-docx-3-new-10.1177_10482911251334843 - Supplemental material for Demonstration of a Home Laundering Method for Cloth Facepieces to Achieve Hygienic and Sustainable Reuse [file sj-docx-3-new-10.1177_10482911251334843.docx]

Figure S2 Overall facepiece performance (with Filter A) after washing at 60^o^C – for specific particle size bins (pre-wash values removed).
