# Supplementary material for: Demonstration of a Home Laundering Method for Cloth Facepieces to Achieve Hygienic and Sustainable Reuse
Source: New Solut. 2025 May 8;35(2):173–87. doi: 10.1177/10482911251334843 (PMC12222837; doi:10.1177/10482911251334843)
Supplement: sj-docx-4-new-10.1177_10482911251334843 - Supplemental material for Demonstration of a Home Laundering Method for Cloth Facepieces to Achieve Hygienic and Sustainable Reuse [file sj-docx-4-new-10.1177_10482911251334843.docx]

**Table S1. Sampling of guidance for the laundering of cloth face coverings intended for reuse as an intervention in the transmission of respiratory diseases.**

| Organization | Guidance | Washing | Drying |
| --- | --- | --- | --- |
| AATCC (American Association of Textile Chemists and Colorists) | Guidance and Considerations for General Purpose Textile Face Coverings: Adult 9 ^42^ | Follow all applicable local and national regulations. | Follow all applicable local and national regulations. |
| AFNOR (French: Association Française de Normalization; English: French Standardization Association) | Barrier masks Guide to minimum requirements, methods of testing, making and use ^60^ | 60°C for 30 minutes |  |
| ANSM (French: Agence nationale de securite du medicament et des produits de sante; English: French National Agency for Medicines and Health Products Safety) | Specifying the treatment protocol [allowing reuse of fabric masks for non-sanitary use provided for in the context of the covid epidemic ^65^ | At home, machine-wash with a standard detergent at 40 degrees, 30 minutes. In commercial laundries, 30 minutes at 60 °C | Machine or air dry |
| ASTM International | F3502 Standard Specification for Barrier Face Coverings ^66^ | As specified by manufacturer. | As specified by manufacturer. |
| BSI (British Standards Institute) | BSI Flex Version 2.1 - Community face coverings – Specification ^67^ | At home: Minimum of 40°C using a normal wash procedure.  Communal laundry facility: 70 °C minimum (as defined by  BS EN ISO 15797) |  |
| CDC (US Centers for Disease Control and Prevention) | Use and Care of Masks ^68^ | Wash by hand or use a washer. | Dry by hand or use a dryer. |
| CEN (European Committee for Standardization) | CEN CWA 17553: 2022 Community face coverings - Guide to minimum  requirements, methods of testing and use (June 2020) ^69^ | 40°C minimum with the use of consumer laundry detergent. 60°C if the use of consumer laundry detergent is not instructed. Duration of washing machine cycle of minimum of 30 minutes (normal settings – not the economic settings) | Follow either the claimed detailed cleaning instructions or the procedure described in EN ISO 6330. |
| GINETEX (International Association for Textile Label Care) | HOW TO CLEAN A FABRIC FACE MASK IN THE TIME OF COVID-19^70^ | 60°C for 30 minutes with a classic laundry soap | 60°C minimum in a dryer (delicate textile).  Natural drying in a clean and ventilated area. |
|  |  |  |  |
| National Health Commission (People’s Republic of China) | Prevention and control program of COVID-19 (6th edition) ^71^ | Heating for a time period of 30 minutes at 56°C. |  |
| WHO (World Health Organization)/ | Infection prevention and control in the context of coronavirus disease (COVID-19): A living guideline 2022 Apr 25 ^72^ | At least 60°C /140°F with soap or detergent  If <60°C/140°F, with soap or detergent followed by boiling for 1 minute |  |
