# Supplementary figures and images for: Demonstration of a Home Laundering Method for Cloth Facepieces to Achieve Hygienic and Sustainable Reuse
Source: New Solut. 2025 May 8;35(2):173–87. doi: 10.1177/10482911251334843 (PMC12222837; doi:10.1177/10482911251334843)

**Table S2. Results of independent testing to EN14683 mean particle size 3um.**


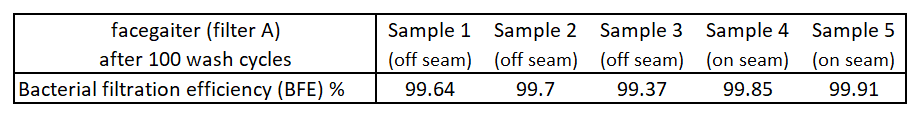

Supplement: sj-docx-5-new-10.1177_10482911251334843 - Supplemental material for Demonstration of a Home Laundering Method for Cloth Facepieces to Achieve Hygienic and Sustainable Reuse [file sj-docx-5-new-10.1177_10482911251334843.docx]

**Table S6. Summary of Leakage Ratios of the facepiece (with Filter B).**


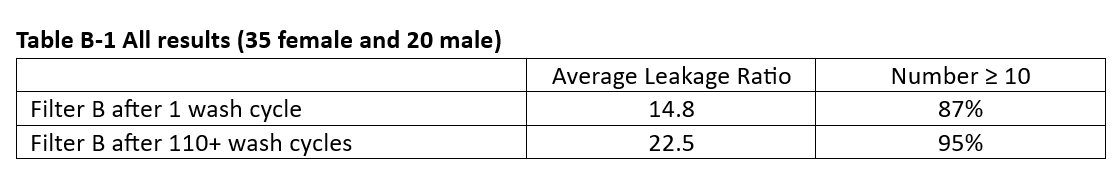

Supplement: sj-docx-9-new-10.1177_10482911251334843 - Supplemental material for Demonstration of a Home Laundering Method for Cloth Facepieces to Achieve Hygienic and Sustainable Reuse [file sj-docx-9-new-10.1177_10482911251334843.docx]
