# Supplementary material for: Demonstration of a Home Laundering Method for Cloth Facepieces to Achieve Hygienic and Sustainable Reuse
Source: New Solut. 2025 May 8;35(2):173–87. doi: 10.1177/10482911251334843 (PMC12222837; doi:10.1177/10482911251334843)

**Table S4. Airflow Resistance of a size medium facepiece (with Filter B), tested over the full area of the filter at 85Lpm after 1 and 50 laundry cycles**


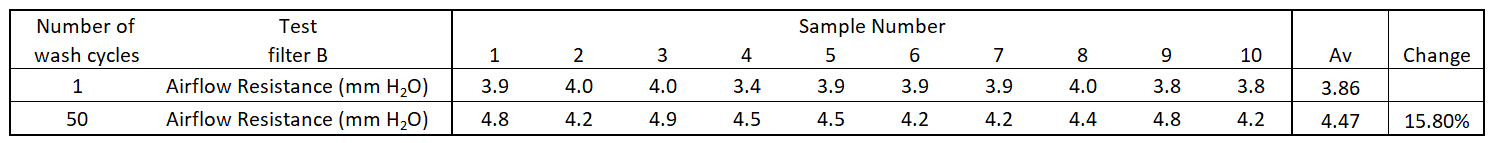

Supplement: sj-docx-7-new-10.1177_10482911251334843 - Supplemental material for Demonstration of a Home Laundering Method for Cloth Facepieces to Achieve Hygienic and Sustainable Reuse [file sj-docx-7-new-10.1177_10482911251334843.docx]
